# Co-adaptive Training Improves Efficacy of a Multi-Day EEG-Based Motor Imagery BCI Training

**DOI:** 10.3389/fnhum.2019.00362

**Published:** 2019-10-14

**Authors:** Amjad Abu-Rmileh, Eyal Zakkay, Lior Shmuelof, Oren Shriki

**Affiliations:** ^1^Department of Cognitive and Brain Sciences, Ben-Gurion University of the Negev, Beer-Sheva, Israel; ^2^Department of Physiology and Cell Biology, Zlotowski Center for Neuroscience, Ben-Gurion University of the Negev, Beer-Sheva, Israel; ^3^Department of Computer Science, Zlotowski Center for Neuroscience, Ben-Gurion University of the Negev, Beer-Sheva, Israel

**Keywords:** brain-computer interface, electroencephalograpy, motor-imagery, machine learning, coadaptation, skill acquisition

## Abstract

Motor imagery (MI) based brain computer interfaces (BCI) detect changes in brain activity associated with imaginary limb movements, and translate them into device commands. MI based BCIs require training, during which the user gradually learns how to control his or her brain activity with the help of feedback. Additionally, machine learning techniques are frequently used to boost BCI performance and to adapt the decoding algorithm to the user's brain. Thus, both the brain and the machine need to adapt in order to improve performance. To study the utility of co-adaptive training in the BCI paradigm and the time scales involved, we investigated the performance of two groups of subjects, in a 4-day MI experiment using EEG recordings. One group (control, *n* = 9 subjects) performed the BCI task using a fixed classifier based on MI data from day 1. In the second group (experimental, *n* = 9 subjects), the classifier was regularly adapted based on brain activity patterns during the experiment days. We found that the experimental group showed a significantly larger change in performance following training compared to the control group. Specifically, although the experimental group exhibited a decrease in performance between days, it showed an increase in performance within each day, which compensated for the decrease. The control group showed decreases both within and between days. A correlation analysis in subjects who had a notable improvement in performance following training showed that performance was mainly associated with modulation of power in the α frequency band. To conclude, continuous updating of the classification algorithm improves the performance of subjects in longitudinal BCI training.

## 1. Introduction

Brain-computer interface (BCI) systems translate brain signals, e.g., electroencephalography (EEG) into control commands for a computer application or a neuroprosthesis. A popular paradigm for BCI communication is motor imagery (MI) (Wolpaw and Wolpaw, 2012; Perdikis et al., 2016; Schultze-Kraft et al., 2017). In this paradigm, the user imagines performing a movement with a particular limb, a process which alters the rhythmic activity in locations in the sensorimotor cortex that correspond to the imagined limb. The BCI system detects these differences and provides the subjects with feedback in the form of cursor movements or other computer commands (Wolpaw and Wolpaw, 2012). Importantly, acquisition of BCI control requires practice, which arguably involves the acquisition of a new representation of the task (Shanechi et al., 2016); that is, using the feedback that the subjects receive, their brains gradually adapt to the task and produce a more effective input signal to the BCI (Wolpaw and Wolpaw, 2012; Alkoby et al., 2017). Apart from the learning of the subjects, learning also takes place within the decoding component by adapting classifier parameters in a way that reduces performance errors (Vidaurre et al., 2011a,b; Perdikis et al., 2016). This classifier adaptation process can occur in parallel with human learning, thereby potentially reducing the amount of practice needed to achieve an effective use for the BCI system.

Practical usage of BCIs has been hindered by their instability. EEG signals are non-stationary due to hardware constraints, such as variations in electrode impedance and positioning from day to day, or due to variability in the cognitive processes that occur during task's performance (such as changes in attention levels, boredom, frustration, etc.). Thus, in addition to the contribution of adaptation for the mutual learning of the brain and the classifier, adaptation has the potential of mitigating the effects of such non-stationarities.

A major goal of the BCI community is to offer potential users, whether disabled or healthy, practical BCI systems that would serve them in the long run, day after day. This paper tackles some issues that are frequently overlooked in EEG-based BCI research, such as the challenges involved in BCI use over multiple days and the limitations of classical training protocols (Lotte et al., 2013; Jeunet et al., 2016). In particular, a major question is to what extent does multi-day BCI training benefit from co-adaptation of the classifier. Classical neurofeedback experiments, which involve no parameter adaption, have shown limited success. To address this shortcoming, in many BCI experiments the classifiers are recalibrated at the beginning of each BCI session, but this procedure is time consuming and may limit the adoption of BCI systems for long-term daily use. Thus, it is important to quantify the contribution of classifier adaptation over using a fixed classifier. To address this question systematically and disentangle the effects of machine learning and human learning, we trained two groups of subjects in a 4-day MI experiment using EEG recordings. During the first day of the experiment, both groups performed four runs followed by classifier adaptation, to ensure a minimal baseline performance level. In the subsequent days, one group performed the BCI task using a fixed classifier based on EEG data from the last two runs on the first day (the control group). In the second group (experimental), the classifier was regularly updated during the experiment days based on the most recent brain activity (last two runs). Importantly, the experimental protocol was identical during the first day, and thus differences in the experimental protocol between the groups started only on the second day. To increase the motivation of subjects in performing the task over many sessions, we designed a video-game training environment, in which movement imagination signals controlled performance in an engaging and rewarding task. In line with previous work on co-adaptation, we used a supervised adaptation paradigm, where the true class labels of the incoming EEG signals are known (Schlögl et al., 2010). Our goal was to quantify the performance difference between the two groups, in order to assess the contribution of continuous classifier co-adaptation compared to using a fixed classifier (which was adapted only on the first day).

The idea of co-learning and co-adaptive training was studied before (Lotte et al., 2018). For example, Vidaurre et al. (2011a,b), show that adaptive LDA classifier enabled some users who were initially unable to control the BCI to achieve better than chance classification performance. Nevertheless, the experimental paradigm included a single session and the advantage of co-adaptive learning compared to a non-adaptive paradigm was not examined. In the present study, we focus on multi-day training and directly compare a co-adaptive protocol with a non co-adaptive protocol.

In a recent work by Muller et al. (2017), a theoretical model of the two-learner problem in BCI was proposed. The results showed that an adaptation that is either too fast or too slow can actually be detrimental to user learning. Therefore, there is a clear need to develop adaptation paradigms that guarantee human learning and boost it. Important issues in this regard are what is the optimal rate for adapting the classifier and under what conditions the mutual learning process will eventually converge to a stable solution (Lotte et al., 2018).

## 2. Methods

### 2.1. Participants

Eighteen healthy volunteers met the inclusion criteria (see below) and participated in this study, twelve females and six males. The age of the participants was 19 to 27 years (Mean = 23.8; *SD* = 1.9). They were all right handed (as verified by observation and as part of the screening questionnaire), had no history of neurological or psychiatric disorders and had normal or corrected-to-normal vision. All participants were naive to BCI, gave written informed consent, and were paid for their participation. The study was approved by a local ethics committee (Ben-Gurion University) and is in accordance with the ethical standards of the Declaration of Helsinki.

### 2.2. Experimental Paradigm

**Figure 1:**
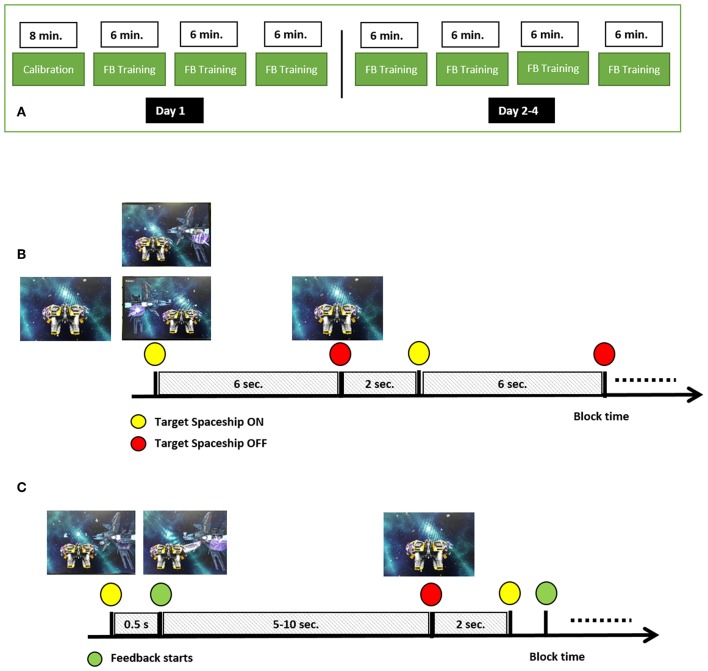
MI-BCI training paradigm. **(A)** Design of the multi-day experiment, **(B)** Calibration run setup, **(C)** Feedback (FB) training runs.

The study consisted of 4 daily sessions performed over 4 consecutive days, each session consisted of four runs. The first session started with a calibration run, while all the following 15 runs were BCI training sessions (Figure 1A). The first run was used for calibration of the BCI system, and served as an opportunity for the participants to get familiar with MI. In each session participants were seated comfortably in an armchair in front of a screen. In the calibration run, the participants were asked to imagine movement of the right hand (RH) and left hand (LH) without getting visual feedback on the screen. At the beginning of the run, a yellow spaceship appeared in the center of the screen, representing the subject-controlled spaceship, navigating through a galaxy (Figure 1B). The run consisted of a block of 60 trials (30 for RH MI, 30 for LH MI). A trial lasted 6 s, with 2 s between trials (see Figure 1B). At the beginning of a trial a cue, in the form of a target spaceship, appeared on the left or right side of the screen, indicating which MI condition the participants should perform, and stayed there for the rest of the trial. After 6 s the target spaceship disappeared and the next target appeared after an inter-trial interval of 2 s. The task of the participants was to perform a kinesthetic MI (imagining squeezing a ball with the left or right hand) as soon as the cue appeared and as long as the target spaceship was on the screen. Participants were asked to focus on the spaceships and avoid producing unnecessary muscle or eye movements, and to restrict movements like eye blinking or swallowing to the inter-trial period.

The setup of BCI training runs was the same as for the calibration run with the exception that 0.5 s after the start of the trial the participants received feedback about the decoding of the BCI algorithm (Figure 1C). Feedback was provided in the form of a laser beam extending out of the personal spaceship toward the target spaceship, with the direction and length of the beam being controlled by the classifier output sign (i.e., negative go to the left, positive go to the right) and amplitude, respectively. Each training run lasted 6 minutes, and the total trial length varied between 5 to 10 s, depending on subject performance; a longer laser beam in the correct direction for a longer period made the target spaceship disappear faster. Thus, the number of trials in each 6-min run was not fixed (mean across participants = 41, *SD* = 12; min = 30, max = 52). The maximal number of trials in a run contained equal numbers of trials for each direction. At the end of each trial, the participant was presented with the number of points that he or she received. The points were added to the total run score. For each trial, the subject received between 0 and 10 points. The given points were inversely proportional to the time required to finish the trial (i.e., range from 0 points for 10 s trials to 10 points for 5 s trials). The video-game training environment and the subject-specific trials were used to increase subject's engagement (Lotte et al., 2012; Jeunet et al., 2016).

### 2.3. EEG Data Acquisition and Processing

The EEG was recorded from 10 Ag/AgCl scalp electrodes arranged in two Laplacian channels located over the sensorimotor cortex (C3, C4) (Wolpaw and Wolpaw, 2012; Yang et al., 2017). The signals were acquired with a g.USBamp amplifier (*Guger* *Technologies*, Austria) with 256 Hz sampling rate, 0.5 Hz high-pass, and 30 Hz low-pass filter, and an additional 50 Hz notch filter. Electrode impedance was regularly checked to ensure impedance below 5 KΩ.

### 2.4. Feature Extraction and Classification

The data of the calibration run of every participant were used to identify subject-specific features. Specifically, the EEG power spectrum of every participant during the calibration run was calculated to obtain subject-specific α and β frequency bands (Yang et al., 2017), which discriminated best between the two MI classes. The α band was defined as a 4 Hz band (±2 Hz around the central frequency), with a central frequency that varied between 8 and 11 Hz. Similarly, the β band was defined as a 6 Hz band (±3 Hz around the central frequency), with a central frequency that varied between 17 and 27 Hz. For each pair of central frequencies, a Linear Discriminant Analysis (LDA) classifier was trained, and the pair that produced optimal classification accuracy was chosen for all subsequent runs.

The band powers of these frequency bands were used as features to set up the LDA classifier in a two-stage process. First, for each 500 ms time window, from the first to the last second within the trials, a classifier was trained and validated by a 10-fold cross-validation. In the second stage, the best detected point in time was used to generate a single classifier, which was subsequently used for providing continuous online feedback to the subject every 500 ms. During the first day, there were three training runs following the calibration run. At the end of each run, a new classifier was trained based only on the last two runs, and was then used in the next run. The difference between experimental days is highlighted in the following section.

### 2.5. Control and Experimental Groups

The 18 participants who met the inclusion criteria were randomly divided into two groups. One group (n = 9; 7 females) performed the BCI training in days 2 to 4 using the classifier that was designed based on the MI data from the last run in day 1 (control group). In the second (experimental) group (*n* = 9; 5 females), the classifier was regularly updated based on brain activity patterns during the experimental days. The classifier update followed a “batch” approach; the two most recent runs were used to create a new classifier, and this new classifier was used in the following run. For the first run in a day, the classifier was constructed based on the last two runs of the previous day. For the second run in a day, the classifier was constructed based on the last run of the previous day with the first run of that day. The accuracy of each run was calculated as the proportion of successful trials (trials in which the target spaceship was destroyed) in each run. This calculation was performed offline at the end of each run.

Subjects were included in the experiment only if their accuracy at the final run of the first day was above 70%. This decision was based on a previous pilot study, in which we found that subjects with very low initial performance often did not improve. In total, 3 subjects (out of an original pool of 21 subjects) with performance lower than 70% were excluded from the experiment after the first day and did not participate in subsequent sessions. At the end of the first day, the groups had comparable performance (Control: mean = 80.1%, *SD* = 8.6%; Experimental: mean = 75.3%, *SD* = 5.6%). One-tail t-test was used to assess the statistical significance of the accuracy differences within and between groups.

## 3. Results

As described in the Methods section, the difference between the experimental designs of the two groups started only on the second day of the experiment. Initial performance, defined as the percentage of accuracy obtained by the end of first day, for all 18 subjects who completed the experiment was above 70% (Mean = 77.7%, *SD* = 7.4%). Frequency bands were selected for each subject separately, and showed variability between subjects, especially in the β band (α band center: Mean = 9 Hz, *SD* = 1.03 Hz, β band center: Mean = 21.9 Hz, *SD* = 3.77 Hz).

### 3.1. Control vs. Experimental Group

**Figure 2:**
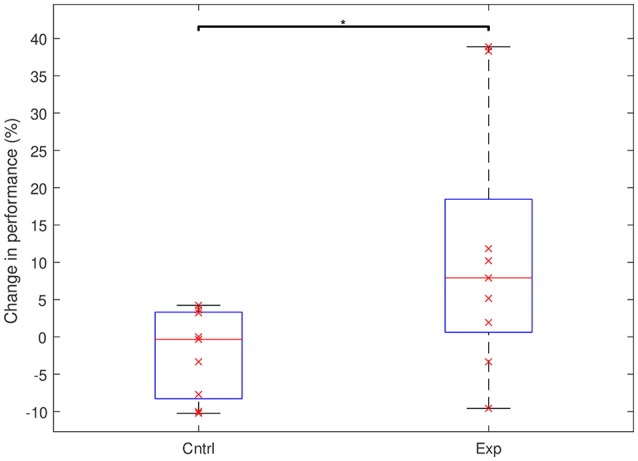
Performance changes in the control and the experimental groups between the start of day 2 and the end of day 4. The groups show a significant difference in behavior (^*^p < 0.05). On each box, the horizontal red line marks the median and the edges of the box are the 25th and 75th percentiles. Whiskers are capped at max. of 1.5 the IQR. The x's represent individual subjects.

**Figure 3:**
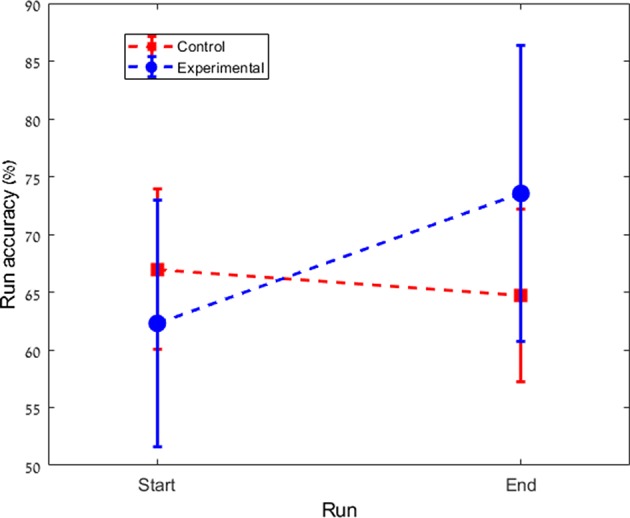
Averaged performance across subjects of each group from the first run of day 2 to the last run of the experiment on day 4 (a total of 12 runs). Co-adapting the classifier boosts the performance of the experimental group.

We found a significant difference in performance changes during training between the experimental and the control groups (Control: Mean = –2.3%, *SD* = 5.8%; Experimental: Mean = +11.3%, *SD* = 16.8%; *p* = 0.023). While in the control group there was no significant change in performance (*p* = 0.136), the experimental group showed a significant improvement across the multi-day experiment (*p* = 0.04). Figure 2 shows the total change in performance for both groups between the beginning of day 2 and the end of day 4. Figure 3 highlights the clear difference in behavior between the groups during the course of the experiment.

### 3.2. Group Dynamics

The groups also exhibit different dynamics of performance during training. In order to study these dynamics we examined changes in performance that occur within a training day and between consecutive training days.

**Figure 4:**
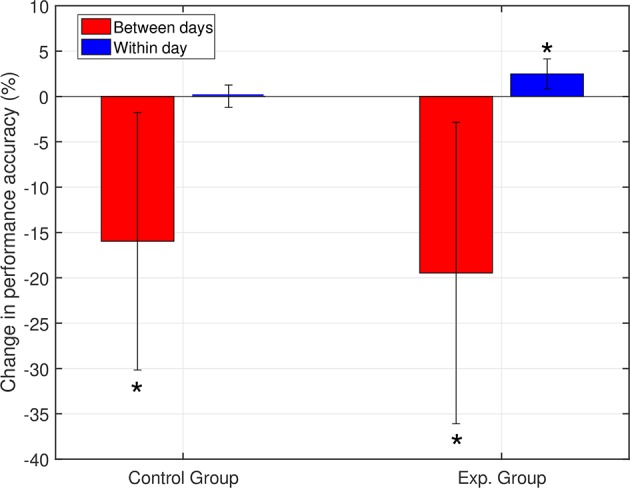
Within- and between-day changes in performance. Both groups showed a decrease between days. For the within-day dynamics, the control group showed no significant change, while the experimental group showed accuracy improvement (^*^p < 0.05). Day 1 was included in calculating the between-day change.

Between-day differences: The between-day change measure represents the mean of all changes between the end of one day and the beginning of the next day in the experiment. For each group, we calculated the grand average of the between-day change across all subjects (including the change between day 1 and day 2). Both groups showed a decrease in the average between-day change in performance (Figure 4), with no statistically significant difference between the groups (*p =* 0.38). The observed decrease in performance between days can be associated with changes in neural dynamics, changes in the recording setup, and changes in the mental and physical state of the subjects.

**Figure 5:**
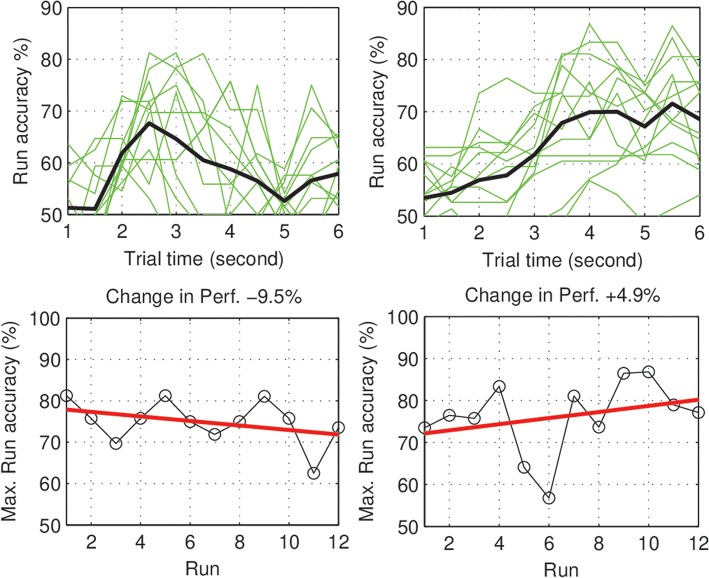
Control subjects (S1, S2): Performance curve per run **(Top)**, and the change in accuracy across the experiment days **(Bottom)**.

**Figure 6:**
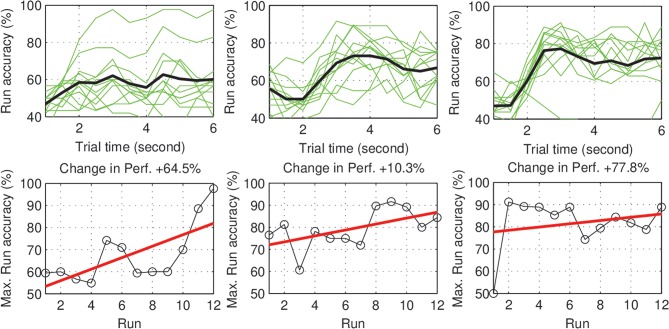
Experimental subjects (S1–S3): Performance curve per run **(Upper)**, and the change in accuracy across the experiment days **(Lower)**. The majority of subjects show a positive trend in performance. The subjects also show different rates (speeds) of improvement.

Within-day differences: to study the within day dynamics in each group, we calculated the average within-day change in performance for each subject. The subject within-day change represents the mean of all changes calculated between two consecutive runs within the same day of the experiment. For each group, the grand average of the within-day change was calculated across subjects. For the control group, the total within-day change showed no significant change in performance (Mean = 0.03%, *SD* = 1.23, with *p =* 0.94), across the experiment period. On the other hand, the experimental group showed a significant within-day improvement (Mean = 2.5%, *SD* = 1.64, with *p =* 0.002) (see Figure 4). To summarize, the incremental within-day improvement in the experimental group compensated for the deterioration caused by the between-day changes, and probably led to an overall increase in performance across the experiment. In the control group, the deterioration due to between-day variations, along with the absence of improvement within days, resulted in an overall decrease in performance. The performance of representative subjects from the control and experimental groups is shown in Figures 5, 6, respectively.

### 3.3. Features-Performance Correlation

To examine the neural features that contributed to the performance in the task, we ran a correlation analysis between the power in each band and the performance across runs. The analysis was conducted only on four subjects from the experimental group who showed robust improvement during training (subjects with 10% or higher improvement in performance during training). Results show that performance in three out of four subjects was associated mainly with modulation of power in the α frequency band (Table 1). Interestingly, power in the α frequency band during resting state was shown to be predictive of BCI performance in a motor imagery task (Blankertz et al., 2010), supporting the suggested functional role of this band in BCI performance.

**Table 1:** Feature-Performance correlation in four subjects from experimental group: for each subject, the feature with maximum correlation with performance is reported.

**Subject**	**Feature**	***r*-value**
S0	α1	0.90
S4	β1	0.35
S6	α2	0.31
S7	α2	0.34

## 4. Discussion

Our results demonstrate that BCI training with an engaging video game and using subject-specific frequency bands is not enough for eliciting improvement in a multi-day BCI training and that improvement with training requires also continuous adaptation of the classification algorithm. We note that in the experimental group, a new classifier was trained after each run based solely on the last two runs. Thus, the training of the new classifier always relied on the same sample size and was not incremental. Improvement in the performance of the experimental group, therefore, cannot be attributed to accumulation of more training data and points to changes in the underlying neural representations of the task, namely in the power within the corresponding frequency bands.

The results indicate that non-stationarity in the brain signal is significant and that on average, subjects cannot overcome its accumulating effect across four days with practice alone. This non-stationarity can be a result of changes in brain signals due to the BCI training. Neurophysiological and imaging studies have repeatedly demonstrated representational changes following the acquisition of a motor skill (Nudo et al., 1996; Hardwick et al., 2013). If indeed the non-stationarity is due to changes in the representation of the task following training, then the BCI paradigm in the context of a fixed classifier is paradoxical, since its acquisition is associated with changes in the brain signals that are needed for its control. Non-stationarity could also result from other sources of variation in the neural signal that are indirectly related to the performance of the subjects. For example, it could change due to changes in the engagement of the subject (Galin et al., 1978). Last, changes in the neuronal patterns could stem from non-task related effects such as variation in the positioning of the EEG electrode, changes in the impedance of the electrodes etc. Such changes are expected to be greater after removing and putting the EEG cap again and are therefore consistent with the between-day changes in performance. Regardless of the source of noise, our results support the idea that adaptation of the readout algorithm is crucial for improving control over BCI. We speculate that with extended training, the performance of subjects that train with a fixed classifier will start to decrease due to increased miss-calibration and frustration. However, it might be that with a longer training period, performance will improve even with a fixed classifier. In addition to decreasing the non-stationarity in the system, updating the classifier may have a secondary effect on the motivation of the subjects; improvement in performance with training increases the engagement of the subjects whereas lack of improvement may lead to frustration and reduced motivation to exercise the control over the BCI system. It also allows subjects to explore different mental strategies to improve the control over their brain rhythms in order to achieve a better performance in the game.

Notably, even within the experimental group some subjects did not show improvement in performance following training, suggesting that updating the classifier is a necessary but not sufficient condition for maintaining and improving BCI performance. This inter-subject variability in terms of the ability to gain and improve control on BCI also suggests that further subject-specific optimization may be needed (e.g., subject specific batch size, identification of invariant features, calibration time, trial duration, and task complexity). The term co-adaptation implies a direct coordination between the human and the machine adaptation processes. It could be that some subjects in the experimental group failed to realize that such a coordination exists in the experiment. In these cases, the simultaneous learning of the human and the machine did not converge and improvement was not attained.

Our results are congruent with recent state-of-the-art BCI protocols that involve feature and classifier calibration, continuous updates of the classifiers (Faller et al., 2012), and an engaging control task (Perdikis et al., 2018). Such protocols have shown the potential of increasing the number of controlled classes (degrees of freedom; McFarland et al., 2010; Friedrich et al., 2013), the proportion of subjects that reach a satisfactory control levels (Faller et al., 2012), and the utility of BCI in real world tasks (Perdikis et al., 2018). Importantly, while these studies have clearly demonstrated a successful acquisition of BCI control, the contribution of each one of the features that are involved in each protocol (such as calibration, adaptation, feedback) to the final performance is lacking. In this study, our aim was to quantify the contribution of classifier's adaptation to the performance of subjects by directly comparing it with respect to a control protocol.

## 5. Conclusions

Many efforts in the BCI field are directed toward designing better features, classifiers and noise reduction techniques (Vidaurre et al., 2011b; Jeunet et al., 2016). Even though these aspects are key to designing a BCI, it is clear from our work that the setting in which the BCI is designed- the training protocol, adaptive disciplines, and considerations of co-adaptive learning - have great influence on the robustness, functionality, and training time of the BCI.

We argue that more work should be focused on co-adaptive learning paradigms and that BCI design processes should take into account that MI BCI is a skill (Shanechi et al., 2016) to be learned by the user, and that user adaptation has great effect on the designed classifier and on the statistics of the changing EEG signals. The ultimate goal is the design of a BCI platform in which brain plasticity and machine learning are combined to achieve a skilled BCI usage for real-life applications. Thus, it would be interesting to pursue experiments with similar paradigms for periods that go beyond four days, to examine long-term convergence of human and machine adaptation.

## Ethics Statement

The study was approved by the local ethics committee of Ben-Gurion University and is in accordance with the ethical standards of the Declaration of Helsinki. All participants gave written informed consent, and were paid for their participation.

## Author Contributions

AA-R, LS, and OS conceived the study and designed the experiments. AA-R and EZ developed the computer codes and performed the experiments. AA-R analyzed the data. AA-R, LS, and OS wrote the manuscript.

### Conflict of Interest

The authors declare that the research was conducted in the absence of any commercial or financial relationships that could be construed as a potential conflict of interest.

## References

[B1] Alkoby O. Abu-Rmileh A. Shriki O. Todder D. (2017). Can we predict who will respond to neurofeedback? a review of the inefficacy problem and existing predictors for successful EEG neurofeedback learning. Neuroscience 378, 155–164. 10.1016/j.neuroscience.2016.12.050 28069531

[B2] Blankertz B. Sannelli C. Halder S. Hammer E. M. Kübler A. Müller K.-R.. (2010). Neurophysiological predictor of smr-based bci performance. Neuroimage 51, 1303–1309. 10.1016/j.neuroimage.2010.03.022 20303409

[B3] Faller J. Vidaurre C. Solis-Escalante T. Neuper C. Scherer R. (2012). Autocalibration and recurrent adaptation: towards a plug and play online erd-bci. IEEE Trans. Neural Syst. Rehabil. Eng. 20, 313–319. 10.1109/TNSRE.2012.2189584 22481835

[B4] Friedrich E. V. Neuper C. Scherer R. (2013). Whatever works: a systematic user-centered training protocol to optimize brain-computer interfacing individually. PLoS ONE 8:e76214. 10.1371/journal.pone.0076214 24086710 PMC3781165

[B5] Galin D. Johnstone J. Herron J. (1978). Effects of task difficulty on eeg measures of cerebral engagement. Neuropsychologia 16, 461–72. 692858 10.1016/0028-3932(78)90069-6

[B6] Hardwick R. M. Rottschy C. Miall R. C. Eickhoff S. B. (2013). A quantitative meta-analysis and review of motor learning in the human brain. NeuroImage 67, 283–297. 10.1016/j.neuroimage.2012.11.020 23194819 PMC3555187

[B7] Jeunet C. Jahanpour E. Lotte F. (2016). Why standard brain-computer interface (BCI) training protocols should be changed: an experimental study. J. Neural Eng. 13:036024. 10.1088/1741-2560/13/3/036024 27172246

[B8] Lotte F. Bougrain L. Cichocki A. Clerc M. Congedo M. Rakotomamonjy A.. (2018). A review of classification algorithms for EEG-based brain computer interfaces: a 10 year update. J. Neural Eng. 15:031005. 10.1088/1741-2552/aab2f2 29488902

[B9] Lotte F. Faller J. Guger C. Renard Y. Pfurtscheller G. Lécuyer A. (2012). Combining BCI with virtual reality: towards new applications and improved BCI, in Towards Practical Brain-Computer Interfaces (Berlin; Heidelberg: Springer), 197–220.

[B10] Lotte F. Larrue F. Mühl C. (2013). Flaws in current human training protocols for spontaneous brain-computer interfaces: lessons learned from instructional design. Front. Hum. Neurosci. 7:568. 10.3389/fnhum.2013.00568 24062669 PMC3775130

[B11] McFarland D. J. Sarnacki W. A. Wolpaw J. R. (2010). Electroencephalographic (eeg) control of three-dimensional movement. J. Neural Eng. 7:036007. 10.1088/1741-2560/7/3/036007 20460690 PMC2907523

[B12] Muller J. Vidaurre C. Schreuder M. Meinecke F. Bunau P. Muller K. (2017). A mathematical model for the two-learners problem. J. Neural Eng. 14:036005. 10.1088/1741-2552/aa620b 28224972

[B13] Nudo R. Milliken G. Jenkins W. Merzenich M. (1996). Use-dependent alterations of movement representations in primary motor cortex of adult squirrel monkeys. J. Neurosci. 16, 785–807. 8551360 10.1523/JNEUROSCI.16-02-00785.1996 PMC6578638

[B14] Perdikis S. Leeb R. d R Millán J. (2016). Context-aware adaptive spelling in motor imagery BCI. J. Neural Eng. 13:036018. 10.1088/1741-2560/13/3/036018 27152498

[B15] Perdikis S. Tonin L. Saeedi S. Schneider C. Millán J. d. R. (2018). The cybathlon bci race: Successful longitudinal mutual learning with two tetraplegic users. PLoS Biol. 16:e2003787. 10.1371/journal.pbio.2003787 29746465 PMC5944920

[B16] Schlögl A. Vidaurre C. Müller K.-R. (2010). Adaptive Methods in BCI Research - An Introductory Tutorial, Berlin, Heidelberg: Springer Berlin Heidelberg, 331–355.

[B17] Schultze-Kraft M. Neumann M. Lundfall M. Wagner P. Birman D. Haynes J.-D. (2017). Predicting Motor Intentions With Closed-Loop Brain-Computer Interfaces. Cham: Springer International Publishing, 79–90.

[B18] Shanechi M. M. Orsborn A. L. Carmena J. M. (2016). Robust brain-machine interface design using optimal feedback control modeling and adaptive point process filtering. PLoS Comput. Biol. 12:e1004730. 10.1371/journal.pcbi.1004730 27035820 PMC4818102

[B19] Vidaurre C. Sannelli C. Blankertz B. (2011a). Machine-learning based co-adaptive calibration: towards a cure for BCI illiteracy. Neural Comput. 23, 791–816. 10.1162/NECO_a_00089 21162666

[B20] Vidaurre C. Sannelli C. Müller K.-R. Blankertz B. (2011b). Co-adaptive calibration to improve BCI efficiency. J. Neural Eng. 8:025009. 10.1088/1741-2560/8/2/025009 21436515

[B21] Wolpaw J. Wolpaw E. W. (2012). Brain-Computer Interfaces: Principles and Practice. Oxford University Press.

[B22] Yang Y. Chevallier S. Wiart J. Bloch I. (2017). Subject-specific time-frequency selection for multi-class motor imagery-based BCIs using few laplacian EEG channels. Biomed. Signal Process. Control 38, 302–311. 10.1016/j.bspc.2017.06.016

